# External Contamination in Single Cell mtDNA Analysis

**DOI:** 10.1371/journal.pone.0000681

**Published:** 2007-08-01

**Authors:** Yong-Gang Yao, Hans-Jürgen Bandelt, Neal S. Young

**Affiliations:** 1 Hematology Branch, National Heart, Lung, and Blood Institute, National Institutes of Health, Bethesda, Maryland, United States of America; 2 Department of Mathematics, University of Hamburg, Hamburg, Germany; University of Glasgow, United Kingdom

## Abstract

**Background:**

Mitochondrial DNA (mtDNA) variation in single hematopoietic cells, muscle fibers, oocytes, and from tiny amount of tumor tissues and degraded clinical specimens has been reported in many medical publications. External DNA contamination, notoriously difficult to avoid, threatens the integrity of such studies.

**Methodology/Principal Findings:**

Employing a phylogenetic approach, we analyzed the geographic origins of mtDNA sequence anomalies observed during multiple studies of mtDNA sequence variation in a total of 7094 single hematopoietic cells. 40 events with irregular mtDNA patterns were detected: eight instances (from seven different haplotypes) could be traced to laboratory personnel; six cases were caused by sample cross-contamination. The sources of the remaining events could not be identified, and the anomalous sequence variation referred to matrilines from East Asia, Africa, or West Eurasia, respectively. These mtDNA sequence anomalies could be best explained by contamination.

**Conclusions:**

Using the known world mtDNA phylogeny, we could distinguish the geographic origin of the anomalous mtDNA types, providing some useful information regarding the source of contamination. Our data suggest that routine mtDNA sequence analysis of laboratory personnel is insufficient to identify and eliminate all contaminants. A rate of 0.6% of external contamination in this study, while low, is not negligible: Unrecognized contaminants will be mistaken as evidence of remarkable somatic mutations associated with the development of cancer and other diseases. The effective contamination rate can increase by a factor of more than an order of magnitude in some studies that did not institute high standards. Our results are of particular relevance to mtDNA research in medicine, and such an approach should be adopted to maintain and improve quality control in single-cell analyses.

## Introduction

Mitochondria provide energy to the cell and have important roles in intracellular signaling and apoptosis and in intermediary metabolism [1], [2]. Each mitochondrion contains many copies of DNA and its own transcriptional and translational machinery. Human mtDNA is a 16.6 kb, double-stranded circular molecule, which encodes 13 oxidative phosphorylation proteins, 22 tRNAs, and 2 ribosomal RNAs [3]. Mitochondrial dysfunction, due to mutations in the nuclear or mitochondrial DNAs, cause a wide range of aging-related diseases as well as specific neurological and auditory syndromes [2], [4], [5].

A high frequency of somatic mtDNA mutations has been reported in various adult post-mitotic cells and in cancer tissues, implicating mtDNA sequence variation in physiological aging and tumorigenesis [2], [4], [6]–[13]. Based on analyses of mtDNA variation in single cells, recent studies have provided deeper insight into the somatic mutation pattern in colorectal mucosa [14], [15], muscle fibers [16]–[21], cardiomyotes [22], hematopoietic cells [13], [23], [24], neurons [25], [26], and the segregation pattern of mtDNA mutation in oocytes and dividing cells [27], [28]. The complete mtDNA genome can be determined in single muscle fibers [21], [29]. However, single cell analyses are technically challenging: the risk of contamination is omnipresent and can result in anomalous results. Recognition and elimination of contaminants and their sources is important to maintain data integrity. Guidelines for ancient DNA protocols [10], [30]–[35] in principle could be suggested for working with very small amount of DNA from single cells or clinical specimens.

Recently, we and others have found that the mtDNA data reported in the medical literature are not free of serious errors [10], [36], [37]. Besides misinterpretation and phantom mutations, the mtDNA sequences reported especially in cancer research [10], may often be plagued with contamination and sample crossover, mainly due to the small amount of DNA available from clinical specimens and insufficient attention to the worldwide mtDNA phylogeny, which can be useful for *a posteriori* data assessment [10], [36]–[40]. In previous studies that have determined the entire mtDNA sequence in order to identify mutations in a small amount of cells in comparison to reference cells and which have identified a correlation between the mutation(s) and biochemical defect [14], [15], the reported mtDNA alterations are highly likely to be real rather than artifactual and the inferences are convincing.

We have utilized single-cell analysis methods for mtDNA mutation to examine aging in the hematopoietic system [24], [41]. Somatic mutations observed in single hematopoietic cells may allow tentative estimates of the number of active hematopoietic stem cells and committed progenitors (HSCs) [23] and the kinetics of HSCs during transplantation (authors' unpublished data), as well as the clonality of leukemic blast cells [13]. Although we have aspired to high standards and have instituted precautions to avoid contamination during our experiments, we still encountered anomalous sequences, and two instances unfortunately went unnoticed in one of our earlier studies (cf. [42]). Therefore, it was of interest to systematically examine all potential contamination events in our recent single-cell studies and to trace their source. Our comprehensive analysis of 40 cells with anomalous mtDNA sequences encountered in analysis of a total of 7094 single cells revealed that only a minority (35%) derived from laboratory personnel or sample cross-contamination. The exact sources for the remaining irregular types with varying matriline origins could not be identified.

To our knowledge, this constitutes the first report concerning mtDNA somatic mutations in cancer and normal hematopoietic cells that systematically (re)analyzed samples that were previously the target of analysis in a laboratory. An earlier attempt [43] performed only ‘cosmetic’ changes in some selected samples (such as JHU_MITO #9) from early studies [44], [45], but remained silent about the most salient case of sample contamination/confusion involving JHU_MITO #12 [42].

## Materials and Methods

### Subjects

We collected hair or blood samples from all 41 current and past laboratory members. Individuals no. 1–5 directly handled the samples and performed the single-cell analysis, whereas the remaining members (no. 6–41) did not physically contact samples for single-cell analysis but shared some bench space and office areas with members no. 1–5 (table 1). The samples for single-cell analysis were obtained in projects on leukemia [13], transplantation, and family analysis (table S1). All healthy donors and patients were recruited with informed consent under protocols approved by the Institutional Review Board of the National Heart, Lung, and Blood Institute (NHLBI, Bethesda), M.D.

**Table 1:** MtDNA sequence variation and haplogroup status in current and past laboratory personnel.

Sample	Sequence variation	Haplo-group	Geographic origin
1	16189-16223-16290-16319-73-152-235-263-292-(523-524)delAC	A	East Asian
2	16147-16173-16223Y-16245-16362-73-191insA-194-199-207-263-489	D4c1a	East Asian
3	16126-16148-16294-16304-16519-73-151-263	T2b	West Eurasian
4	16311-146Y-263-480	H	West Eurasian
5	16154-16311-263-455insT	H	West Eurasian
6	16126-16362-16519-57insC-64-263	R0a	West Eurasian
7	16129-16181A/C-16182A/C-16183A/C-16189-16217-16261-16292-16301-16519-61-62-73-263-(523-524)delAC	B4a	East Asian
8	16126-16187-16189-16223-16264-16270-16278-16290-16293-16311-16519-73-150-152-182-184-185C-188-247-263-357-515	L1b	Sub-Saharan African
9	16126-16294-16296-16304-16519-73-263-321	T2b	West Eurasian
10	16172-16219-16235-16278-16355-16519-73-146-263	U6a1	North African
11	16069-16126-73-151-152-185-228-263-295-462-489	J1c	West Eurasian
12	16124-16183A/C-16189-16223-16278-16362-16527-73-263-(523-524)delAC	L3b	Sub-Saharan African
13	16129-16223-16264-16265C-16518C-16519-73-189R-263-489-(523-524)delAC	M5a	South Asian
14	16168-16224-16311-16519-73-185-263-497	K1a	West Eurasian
15	16129-16192-16223-16234-16362-73-153-195-263-489-(523-524)delAC	D4	East Asian
16	263	H2b	West Eurasian
17	16217-73-152-195-263	HV2	West Eurasian
18	16114A-16126-16187-16294-16296-16324-16519-73-152-263	T2	West Eurasian
19	16224-16248-16311-16319-16463-16519-73-152-263-(523-524)insAC	K1b1a	West Eurasian
20	16174-16223-16311-16362-73-152-263-489-(523-524)delAC	D4	East Asian
21	16150-16182C-16183C-16189-16311-152-195-263-270	H11	West Eurasian
22	16189-16192-16223-16278-16294-16304-16309-16390-73-146-152-195-263-534	L2a1	Sub-Saharan African
23	16048-16129-16218-16223-16301-16357-55insT-57-59-73-194-263-489-(523-524)delAC	M5c	South Asian
24	16519-263	H	West Eurasian
25	16270-16519-263	H	West Eurasian
26	16311-93-263	H	West Eurasian
27	16519-263	H	West Eurasian
28	16126-16163-16186-16189-16294-16519-73-152-195-263	T1a	West Eurasian
29	16129-16145-16223-16297-73-150-263-372-489	M7b	East Asian
30	16162-16172-16209-16291-16519-73-263	H1a	West Eurasian
31	16092-16223-16319-16356-16362-73-263-489-(523-524)delAC	D4b	East Asian
32	16093Y-16129-16189-16278-16300-16354-16390-16399-16519-73-146-150-189-195-263-456-573+C	L2d	Sub-Saharan African
33	16140-16182C-16183C-16189-16266A-16519-73-210-263-(523-524)delAC-593	B5a	East Asian
34	16140-16183C-16189-16234-16266A-16519-73-150-210-263-(523-524)delAC	B5a	East Asian
35	16263-16519-263-477	H1	West Eurasian
36	16126-16294-16296-16304-16519-73-263	T2b	West Eurasian
37	16183C-16189-16300-16304-16311-16519-73-150-195-249delA-263-(523-524)delAC-573insCCCCC	F1b	East Asian
38	16223-16320-16355-16519-73-150-195-263	L3e2	Sub-Saharan African
39	16189-16292-16304-16497-16519-73-263-373	R30	South Asian
40	16145-16176-16224-16233-16311-16456-16519-16527-73-263	K	West Eurasian
41	16093Y-16311-16519-263	H	West Eurasian

Note: Sequence variation of each individual was determined from a single hair (with the exception of samples 10 and 22 in which genomic DNA from whole blood was used) and was scored relative to the Cambridge Reference Sequence (CRS) [3]. Suffixes Y and A/C meant heteroplasmy for C and T, A and C, at the respective site. Suffixes A and C indicated transversions; “ins” and “del” indicated insertions and deletions, respectively. Indels (insertion/deletion) were recorded at the last possible site. Length mutations of the C-tract in regions 16184–16193 and 303–309 were not included. All samples had 315+C in the second hypervariable segment of mtDNA control region. The haplogroup status of the samples and geographic origin of the haplogroups were estimated according to the world mtDNA phylogeny as known to date [46]–[51], [54].

### Genomic DNA extraction

Genomic DNA from whole blood samples was extracted by using QIAamp DNA Mini Kit (Qiagen) according to the manual of the manufacturer. We plucked a single hair and instantly digested the hair root (containing follicular sheath material) in 50 µL of lysis buffer (10 mmol/L Tris-HCl [pH 8.0], 50 mmol/L KCl, 100 µg/mL Proteinase K, 1% Triton X-100) at 56°C for 1 h, then the lysate was incubated at 96°C for 8 min to inactivate the proteinase K. The procedure for single-cell DNA extraction has been described in detail in our recent studies [13], [23]. In brief, mononuclear cells from peripheral blood were separated by Ficoll density gradient centrifugation, washed, and stained with respective antibodies. Single cells were sorted into 96-well plates by flow cytometry and were lysed in 50 µL lysis buffer/well.

### mtDNA amplification and sequencing

PCR amplification of single cells was performed in 96-well plates using the same procedure and condition described in our recent study [13]. In brief, 5 µL of cell lysates were amplified in 30 µL of reaction mixture containing 400 µM of each dNTP, 1 units of TaKaRa LA Taq^TM^ , which has proof reading activity (Takara Bio. Inc.), 0.5 µM of each forward and reverse outer primer (L15594: 5′-CGCCTACACAATTCTCCGATC-3′ and H901: 5′-ACTTGGGTTAATCGTGTGACC-3′). The amplification was run on the GeneAmp PCR system 9700 (Applied Biosystems, Foster City, CA) with one denaturation cycle of 94°C for 3 min, then 35 cycles of 94°C for 30 sec, 52°C for 40 sec and 72°C for 1 min with a 5 sec increase per cycle, and ending with a full extension cycle of 72°C for 10 min. The second PCR was performed in 50 µL of reaction mixture containing 400 µM of each dNTP, 2 units of TaKaRa LA Taq^TM^, 0.5 µM of each forward and reverse inner primers (L15990: 5′-TTAACTCCACCATTAGCACC-3′ and H650: 5′-GAAAGGCTAGGACCAAACCTA-3′), and 5 µL of first PCR product. Amplification condition for the second PCR included one denaturation cycle of 94°C for 3 min, then 35 cycles of 94°C for 30 sec, 52°C for 40 sec, and 72°C for 90 sec, ending with a full extension cycle of 72°C for 10 min.

Second PCR products were purified using the QIA quick PCR purification kit (Qiagen, Valencia, CA) or the ExcelaPure 96-Well UF PCR Purification Kit (EdgeBiosystems, Gaithersburg, MD). The purified PCR products were overlap sequenced using the inner primers (L15996, 5′-CTCCACCATTAGCACCCAAAGC-3′; L16209, 5′-CCCCATGCTTACAAGCAAGT-3′; L16517, 5′-CATCTGGTTCCTACTTCAGG-3′; H26, 5′-GCATGGAGAGCTCCCGTGAGTGG-3′; L29, 5′-GGTCTATCACCCTATTAACCAC-3′; L332, 5′-CCCGCTTCTGGCCACAGCAC-3′) and primer H650, as described in our recent study [13]. Sequencing was performed by using BigDye Terminator v3.1 Cycle Sequencing Kit and was run on a 3100 DNA sequencer (Applied Biosystems) according to the manufacturer's manual.

The DNA samples extracted from whole blood (100 ng) and hair (2 µL of lysate) were amplified by using primer pair L15990/H650 and the same conditions as the second PCR for single cells. PCR products were purified by using QIAamp Gel Extraction Kit and were sequenced in the same way as for single cells.

To discern any reagent contamination and to validate our technique, we performed two-step PCR in two 96-well plates, which included the PCR reaction mixture but replaced cell lysate or first PCR product with ddH_2_O, and followed the same procedure as for single cells. We observed no amplification in any of the 192 wells, indicating absence of contamination of reagents. In each amplification of single cells from a sample, we also included one to four negative controls.

### mtDNA anomaly recognition and phylogenetic analysis

**Figure 1:**
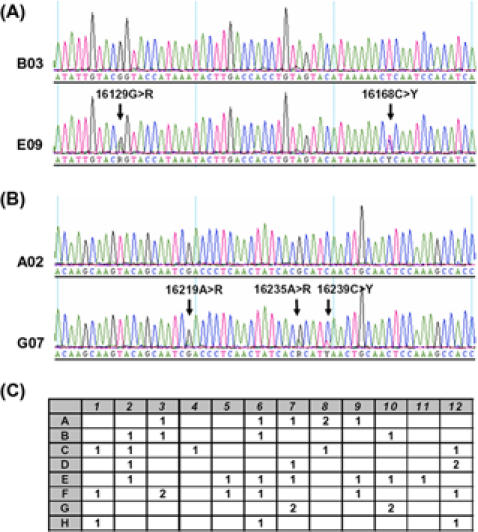
Recognition of mtDNA sequence anomalies in single cell analysis. Sections of representative sequence electropherograms showing external DNA contamination in a single cell from samples A-2 (A) and donor 7 (B), together with an uncontaminated cell from the respective sample. Sites with heteroplasmic mutations (R for A and G; Y for C and T) were scored relative to the Cambridge Reference Sequence [3]. (C) Occurrence of contamination events in the wells of the 96-well plate in analyzing 7094 single cells. The numbers in the wells indicated the number of contamination instances observed in the respective well. Wells with no contamination were left empty.

We followed a simple approach to pinpoint anomalous mtDNA sequence in single cells. A cell showing mutations at nearly all variable sites of the aggregate mtDNA sequence from the individual's sample was regarded as anomaly or contamination (figure 1A, B). The sequence variation of the irregular mtDNA could be read from all the observed variable sites by filtering those sites that were present in the aggregate sequence of the single cells (table S1). Such a filtering strategy places the potential somatic mutation(s) in the contaminated cell into the inferred sequence of the contaminant and cannot distinguish the shared heteroplasmic mutations between the contaminant and the cell. In several cases, the determination of a cell (lysate from certain well in a 96-well plate) yielded a sequence different from the aggregate sequence of single cells from the same sample by many homoplasmic mutations, and the sequence variation of this mtDNA type was directly read from the determined sequence. All mtDNA sequence types of laboratory personnel, samples for single-cell analysis, and contaminants, were classified relative to the available world mtDNA phylogeny [46]–[51]. The ultimate geographic origins of the matrilines were inferred according to the current continental distribution of the haplogroups (which are monophyletic clades in an mtDNA tree). In this context, the phylogenetic method was used to discern the potential origin of the mtDNA sequence anomalies rather than to identify spurious genetic alterations in our single-cell analysis; the phylogeographic information thus provides an explanation for some of the observed sequence anomalies.

## Results

### Haplogroup classification of mtDNAs

The classification of 41 mtDNAs from laboratory personnel revealed various continental sources for these matrilines and was in general agreement with the claimed geographic origin, with the exception of two members carrying mtDNAs from haplogroups of African provenance (table 1). Ten mtDNAs belonged to eight East Asian haplogroups (A, D4c1a, D4, D4b, M7b, B4a, B5a, and F1b) and three mtDNAs to South Asian haplogroups (M5a, M5c, and R30). Six mtDNAs belonged to African-specific haplogroups (L1b, L2a1, L2d, L3b, L3e2, and U6a1). The remaining 22 mtDNAs were assigned to the current West Eurasian mtDNA pool. Two unrelated lab members (24 and 27) shared the same (frequent) haplotype belonging to haplogroup H (table 1). The sample group for single-cell analysis contained 18 matrilines of West Eurasian origin, eight matrilines of potential Native American ancestry, and six matrilines of (North or sub-Saharan) African origin (mtDNAs of the maternally related samples were counted as one matriline; table S1).

### Recognition of mtDNA sequence anomalies

In total, we identified 40 cells with anomalous sequence variation from a total of 7094 single cells by direct sequence comparison, although the negative controls included in these experiments consistently yielded no detectable amplification on 1.5% agarose gel. The frequency of cell showing anomalous sequence was thus low (0.6%). Seven members [including three (members 1, 2, and 5) who handled samples and performed single-cell analysis and four members (members 8, 15, 17, and 24 or 27) who shared some bench space and office areas but did not work with the samples in this study] shared sequence with the anomalous types in eight cells, thus suggesting contamination. Among them, contamination of East Asian origin from member 15 (haplogroup D4) occurred twice. In the two contamination cases caused by members 8 and 17, the determination of the cell lysate showed the exact sequences of the two members and the sequence of the original cell could not be recognized (table S1). Sample cross-contamination, which is common in the field [10], [52], was observed in six cells from two samples with haplogroup status A2 (CB-1; contaminated by sample CB-2) and J1c (UPN16; contaminated by sample UPN18 or ERR or UPN20), respectively. The contaminant sequences of the remaining 26 cells did not match any laboratory member, and the inferred haplogroup status showed that these matrilines were ultimately of East Asian, sub-Saharan African, Native American, or West Eurasian origin. Two contaminant types of West Eurasian ancestry, 16129-73-263-(523-524)delAC and 16239-16519-263, were observed frequently (eleven and six times, respectively); one contaminant with East Asian D5a2a status occurred twice (table S1). The source of these three relatively frequent contaminants remains enigmatic. Note that the above inferred contamination from laboratory members and/or samples determined in the same study might also be contributed by unknown individuals, who by chance shared identical sequences. That the possibility of such an occurrence was very low is suggested by our finding only two laboratory members sharing identical sequence variation among a total of 41 individuals.

We further located the contaminated cells on the 96-well plate, to discern whether some wells were more preferentially subjected to contamination. The occurrence of contamination on the plate did not show any trend (figure 1C), suggesting that the accidental entry of external contaminant DNA into a well during experiments was randomly distributed. Reamplification and resequencing of the original cell lysates in some cells showing anomaly (about 10%) failed to reproduce the anomalous sequence and the newly determined sequence had the consensus sequence expected for most single cells stemming from the sample. Using the original first PCR products of these cells as the template and re-performing the second PCR and sequencing, we consistently detected the anomalous sequence. This result suggests that contamination in these cells occurred during the initial PCR amplification process and not in the pre-PCR stage, or that the potential contaminant DNA was obscured by preferential amplification of the authentic mtDNA in the cell in the independent verification experiment.

## Discussion

Examining mtDNA mutations at the single-cell level has been valuable in understanding cell origin and clonality, the potential mechanism regarding the occurrence and fixation of an mtDNA mutation by random genetic drift or clonal expansion, and the mtDNA mutation process in tumor and post-mitotic cells with age [13]–[15], [22], [23], [53]. However, one frequently encountered problem with single-cell and other analyses for mtDNA utilizing tiny amounts of DNA is contamination, which remains either unrecognized or unacknowledged in the medical literature. With the available information about the world mtDNA phylogeny at hand [46]–[51], [54], we could easily distinguish the broad geographic origin of the anomalous mtDNAs and thus obtain direct evidence of contamination. For instance, with the new data from the present study, the extraordinary mtDNA mutation pattern observed in one of the two CD34^+^ cells in [24] could be resolved: the contaminant DNA present in the bone marrow donor (BM donor 2, U6a1) turned out to be the haplogroup D4c1a type matching the mtDNA of member 2, who handled the sample and performed the analysis at the time [24]. This D4c1a type was also detected in one cell from sample UPN18 that had a Native American matrilineal ancestry in the current study (table S1). In the second instance from [24], the cord blood donor mtDNA (CB donor 1) could be classified as haplogroup M30c, which is specific to South Asian populations [49]. The anomalous sequence found in one CD34^+^ cell from this donor has haplogroup status M39 (based on the shared mutations 153, 463 and 485 with two Indian M39 lineages from [49]), which is also exclusively South Asian. This contaminant type was not found among current and past laboratory personnel and its exact source remains unknown. In the present large-scale study, two characteristics were discerned in the 40 investigated instances: first, the source of 65% of contamination events could not be explained, as the contaminant sequences did not match known sequences of either laboratory personnel or other patient samples involved in the study and three enigmatic contaminants occurred more than once; second, the risk of contamination from personnel who did not directly contact the sample was even higher than for those who handled samples and performed experiments. The story about the “flying” contaminant DNA is thus more complicated than we had thought. Fortunately, the overall rate of external contamination observed in this study was low and true somatic mutations could be detected in single cells [13], [23] or single cell colonies [24]. In those reports that have analyzed the entire mtDNA sequence mutation from a small amount of cells, such as individual and partial cytochrome *c* oxidase-positive or deficient crypts or muscle fibers [14], [15], [21], the reported mtDNA alterations appear valid and to correlate with the biochemical deficiency.

We believe that the observed anomalous mtDNA types are best explained by contamination rather than potential paternal leakage, which has only been reported in a single case to date [55], [56]. First, paternal leakage could not account for the three enigmatic types that were observed multiple times in single cells from different individuals. Second, we failed to obtain the anomalous mtDNA type in some cells when using the original cell lysate as the template in an independent assay, which suggested that it was most likely introduced during the amplification process. Third, in a review by one of us of published instances of putative paternal leakage, based on phylogenetic criteria, contamination appeared to be the more likely explanation for the presence of anomalous sequences [42].

Our approach cannot distinguish a contamination event if the contaminant and the sample show nearly identical sequences (of the same haplogroup status). For example, in sample UPN19 that has sequence variants 16519-146-263, contaminant DNA from laboratory members with the same haplogroup H status (members 16, 24, 26, 27, 35, and 41) and some samples determined in the same study (donor 8) could not be distinguished by the phylogenetic method because of the paucity of distinctive mutations; the heteroplasmic mutations observed in the contaminated cells would be naturally regarded as somatic events. In addition, cross-contamination between different cells of the same sample is virtually impossible to detect. We fully agree with the conclusions from recent ancient DNA studies emphasizing that standard precautions for avoiding contamination are insufficient to eliminate all contamination [34], [35]. In some rare cases, even PCR reagents could be a source for external DNA contamination [57]. Concerning the question of to what extent we can eliminate contamination in single-cell analysis, there is no definitive answer – but a phylogenetic approach as employed here is nevertheless a useful tool to define the geographic origin of the mtDNA sequence anomalities. The phylogenetic method can therefore signal caution in instances in which repeated direct sequence comparisons appear to have confirmed the authenticity of observed results; indeed, there is no guarantee that re-analysis should not suffer from the same systematic error or persistent contamination as in the initial analysis.

The rate of 0.6% of external contamination observed in this study while low is not negligible. Unrecognized contamination will ordinarily be mistaken as evidence towards multiple somatic mutations and may be associated with the development of diseases, like the late stage of cancer. The effective contamination rate can increase by a factor of more than an order of magnitude in some studies, especially when the researchers did not realize the risk of contamination and maintain a stringent data control. Indeed, we recently failed to repeat a high rate of mtDNA somatic mutation in early stage breast cancer under stringent data quality control [58]. It is not helpful when clear instances of sample confusion or contamination – discovered through *a posteriori* analysis – get subsequently defended as “real” somatic effects by unconvincing arguments. For example, in a commentary paper on PLoS Medicine [59], the authors obviously misunderstood the mtDNA information about Patient 2 from Kirches *et al*. [60], who codified mutations not with respect to the reference sequence but contrasted the nucleotides found in the mtDNAs of glioblastoma and of corresponding blood sample; the conclusion of Salas *et al*. [10] that two mtDNAs from different individuals, one from haplogroup J1c1 and the other from a particular branch of haplogroup U4a, could be attributed to this patient is logical. Unfortunately, recent review articles (e.g. [11], [12], [61]) follow the tradition of early reviews (e.g. [62], [63]) in uncritically listing reported results and “pioneer” articles (such as [44]) now known to have severely suffered from artifacts. Nor is it encouraging to see recent recommendations for future research [64] blind to the problems that have plagued the field of mtDNA analysis in cancer research.

## Supporting Information

Table S1 mtDNA sequence anomalies identified in single-cell analysis.
(0.12 MB DOC)
