# Supplementary material for: External Contamination in Single Cell mtDNA Analysis
Source: PLoS One. 2007 Aug 1;2(8):e681. doi: 10.1371/journal.pone.0000681 (PMC1930155; doi:10.1371/journal.pone.0000681)
Supplement: Table S1 — mtDNA sequence anomalies identified in single-cell analysis. (0.12 MB DOC) [file pone.0000681.s001.doc]

Table S1. mtDNA sequence anomalies identified in single-cell analysis

| **Sample** | **Sequence variation** | **Haplo-group** | **Geographic origin** | **No. of cells** | **No. of contaminated cells** | **Anomalous sequence ^a^** | **Contaminant haplogroup** | **Source** | **Geographic origin** | **Location in 96-well plate** |
| --- | --- | --- | --- | --- | --- | --- | --- | --- | --- | --- |
| donor 7 | 16172-16219-16235-16278-16355-16519-73-146-263 | U6a1 | North African | 95 | 2 | 16172Y-16219R-16235R-**16239**Y-16278Y-16355Y-**16519**C-73R-146Y-**263**G | H | unknown | West Eurasian | B06, G07 |
| donor 8 | 16169-16299-16519-263 | H | West Eurasian | 95 | 1 | **16111**Y-16169Y-**16183**A/C-**16189**Y-**16217**Y-16299R-**16483**R-**16519**C-**73**R-**263**G-**306**Y-**499**R | B2 | unknown | Native American | D07 |
|  |  |  |  |  | 1 | 16169Y-**16239**Y-16299R-**16519**C-**103**R-**263**G-**427**Y | H | unknown | West Eurasian | G10 |
| donor 9 | 16111-16189-16224-16256-16311-16519-56insC-58-73-263-315+C-497-(523-524)insAC | K1a | West Eurasian | 94 | 1 | 16111Y-**16129**R-16189Y-**16192**Y-**16223**Y-16224Y-**16234**Y-16256Y-16311Y-**16362**Y-16519Y-56non-insC/InsC-58Y-**489**Y-497Y-**(523-524)delAC**/insAC | D4 | from member 15 | East Asian | F06 |
| BURDC | 16111-16189-16224-16256-16311-16519-56insC-58-73-263-315+C-497-(523-524)insAC | K1a | West Eurasian | 189 | 0 | — | — | — | — | — |
| donor 10 | 16298-16519-72-263 | HV0 | West Eurasian | 95 | 1 | **16092**Y-**16172**Y-**16189**Y-**16223**Y-**16266**Y-16298Y-**16362**Y-16519Y-72Y-**73**R-**150**Y-**263**G-**489**Y-**(523-524)delAC**/non-del | D5a2a | unknown | East Asian | F01 |
| GREAC | 16298-16519-72-263 | HV0 | West Eurasian | 191 | 0 | — | — | — | — | — |
| donor 2 | 16223-16278-16294-16390-73-146-152-195-263-(523-524)delAC | L2a | Sub-Saharan African | 96 | 0 | — | — | — | — | — |
| UPN19 | 16519-146-263-309+CC-315+C | H | West Eurasian | 269 | 0 | — | — | — | — | — |
| UPN20 | 16111-16209-16223-16290-16293C-16319-16362-16519-64-73-146-153-235-263-(523-524)delAC | A2 | Native American | 179 | 1 | 16179delC-16223T-16519C-73G-195A-263G-489C-(523-524)delAC | M30d |  | South Asian | C12 |
|  |  |  |  |  | 1 | 16217C-73G-152C-195C-263G | HV2 | from member 17 | West Eurasian | D12 |
| UPN16 | 16069-16126-73-185-228-263-295-462-482-489 | J1c | West Eurasian | 94 | 2 | 16069Y-**16126**C-**16223**Y-**16290**Y-**16319**R-**16335**R-**16362**Y-**16527**Y-**64**Y-**73**G-**146**Y-**153**R-185R-228R-**235**R-**263**G-295Y-462Y-482Y-489Y-**(523-524)delAC**/non-del | A2 | from UPN18 or ERR or UPN20? | Native American | G10, E11 |
| UPN17 | 16183C-16189-16223-16278-16519-73-153-189R-195-225-226-263 | X2b | West Eurasian | 81 | 1 | 16183A/C-16189Y-16223Y-**16239**Y-16278Y-**16519**C-73R-153R-189R-195Y-225R-226Y-**263**G | H | unknown | West Eurasian | D02 |
| UPN18 | 16086-16111-16223-16290-16319-16362-64-73-146-153-235-263-(523-524)delAC | A2 | Native American | 94 | 1 | 16086Y-16111Y-16223Y-**16239**Y-16290Y-16319R-16362Y-**16519**Y-64Y-73R-146Y-153R-235R-**263**G-(523-524)delAC/non-del | H | unknown | West Eurasian | A08 |
|  |  |  |  |  | 1 | 16086Y-16111Y-**16147**Y-**16173**Y-**16223**T-**16245**Y-16290Y-16319R-**16362**C-64Y-**73**G-146Y-153R-**191+A**/non-ins-**194**Y-**199**Y-**207**R-235R-**263**G-(523-524)delAC/non-del-**489**Y | D4c1a | from member 2 | East Asian | F03 |
| UPN21 | 16223-16325-16362-73-200-263-489 | D1 | Native American | 106 | 0 | — | — | — | — | — |
| UPN1 | 16126-16292-16294-16519-73-146-152-263-279 | T2 | West Eurasian | 147 | 1 | 16126Y-**16224**Y-16292Y-16294Y-**16311**Y-**16519**C-**73**G-**146**C-**152**C-**263**G-279Y-**512**C/A | K2a2a | unknown | West Eurasian | A09 |
| UPN2 | 16069-16126-73-185-188-228-234-260-263-295-462-489 | J1c | West Eurasian | 96 | 0 | — | — | — | — | — |
| UPN3 | 16093-16129-16519-152-263 | H | West Eurasian | 90 | 1 | 16093Y-16129R-**16239**Y-**16519**C-152Y-**263**G | H | unknown | West Eurasian | G07 |
| AGM | 16182C-16183C-16189-16217-16482-16519-73-263-499 | B4b | East Asian / Native American | 96 | 0 | — | — | — | — | — |
| MFSS | 16189-16223-16245-16278-16294-16309-16390-73-143-146-152-263 | L2a1 | Sub-Saharan African | 96 | 0 | — | — | — | — | — |
| LAA | 16362-16482-16526-239-263 | H | West Eurasian | 96 | 1 | **16189**Y-**16223**Y-**16290**Y-**16319**R-16362Y-16482R-16526R-**73**R-**152**Y-**235**R-239Y-**263**G-**292**Y-**(523-524)delAC**/non-del | A | from member 1 | East Asian | F12 |
| JCS | 16038-16209-16223-16294-16311-16519-73-146-263-374insA | L3f | Sub-Saharan African | 96 | 0 | — | — | — | — | — |
| DC | 16223-16278-16286-16294-16309-16390-16519-73-146-152-195-263 | L2a | Sub-Saharan African | 96 | 0 | — | — | — | — | — |
| OAM | 16223-16260-16325-16362-16519-73-152-183-263-489 | D1 | Native American | 190 | 1 | **16129**R-**16192**Y-**16223**T-**16234**Y-16260Y-16325Y-**16362**C-16519Y-**73**G-152Y-**153**R-183R-**195**Y-**263**G-**489**C-**(523-524)delAC**/non-del | D4 | from member 15 | East Asian | C04 |
|  |  |  |  |  | 1 | **16140**Y-**16182**A/**C**-**16183**A/**C**-**16189**Y-16223Y-16260Y-**16274**R-16325Y-**16335**R-16362Y-**16519**C-**73**G-**146**Y-**150**Y-152Y-183R-**195**Y-**263**G-489Y | B4c1b | unknown | East Asian | C08 |
| EMB | 16223-16239-16288-16325-16362-73-210-228-263-489 | D1 | Native American | 95 | 0 | — | — | — | — | — |
| ERR | 16111-16126-16223-16259-16290-16319-16327-16362-16519-73-146-153-235-263-(523-524)delAC | A2 | Native American | 94 | 0 | — | — | — | — | — |
| HERJC | 16189-16519-263-593G | H | West Eurasian | 94 | 1 | **16154**Y-16189Y-**16311**Y-16519Y-**263**G-**455insT**/non-ins-593T/G | H | from member 5 | West Eurasian | H01 |
| ANDGC | 16189-16519-263-593G | H | West Eurasian | 276 | 2 | **16129**R-16189Y-16519Y-**73**R-**263**G-**(523-524)delAC**/non-del-593T/G | ? | unknown | West Eurasian | B10, E05 |
| QUIDC | 16093-16126-16294-16296-16304-16519-73-263-573insC | T2b | West Eurasian | 92 | 1 | **16092**Y-16093Y-16126Y-**16164**R-**16172**Y-**16182**A/C-**16183**A/C-**16189**Y-**16223**Y-**16266**Y-16294Y-16296Y-16304Y-**16362**Y-16519Y-**73**G-**150**Y-**263**G-**489**Y-**(523-524)delAC**/non-del-573insC/non-ins | D5a2a | unknown | East Asian | H12 |
| MAUWC | 16093-16126-16294-16296-16304-16519-73-263-573insC | T2b | West Eurasian | 184 | 0 | — | — | — | — | — |
| KIRGC | 16069-16126-16145-16172-16222-16261-73-242-263-295-462-489 | J1b | West Eurasian | 192 | 1 | 16069Y-16126Y-**16136**Y-16145R-16172Y-**16182**A/C-**16183**A/C-**16189**Y-**16217**Y-16222Y-16261Y-**16325**Y-**16519**Y-**73**G-**207**R-242Y-**263**G-295Y-**454**Y-462Y-489Y-**499**R-**(523-524)delAC**/non-del | B4b1a | unknown | East Asian | F09 |
|  |  |  |  |  | 1 | 16069Y-16126Y-**16129**R-16145R-16172Y-16222Y-16261Y-**73**G-242Y-**263**G-295Y-462Y-489Y-**(523-524)delAC**/non-del | ? | unknown | West Eurasian | C02 |
| THOMC | 16069-16126-16145-16172-16222-16261-73-242-263-295-462-489 | J1b | West Eurasian | 188 | 0 | — | — | — | — | — |
| CABO | 16172-16183C-16189-16223-16320-16519-73-150-152-195-198-263 | L3e2b | Sub-Saharan African | 188 | 1 | 16126C-16187T-16189C-16223T-16264T-16270T-16278T-16290T-16293G-16311C-16519C-73G-150T-152C-182T-184A-185C-188G-247A-263G-357G-515G | L1b | from member 8 | Sub-Saharan African | A07 |
|  |  |  |  |  | 1 | **16165**R-16172Y-16183A/C-16189Y-**16222**Y-16223Y-**16256**Y-**16270**Y-16320Y-**16399**R-16519Y-**73**G-150Y-152Y-195Y-198Y-**263**G | U5a1 | unknown | West Eurasian | E02 |
|  |  |  |  |  | 1 | **16069**Y-**16126**Y-16172Y-16183A/C-16189Y-**16193**Y-16223Y-**16309**R-16320Y-16519Y-**73**G-150Y-152Y-195Y-198Y-**263**G-**295**Y-**462**Y-**489**Y | J1 | unknown | West Eurasian | A06 |
| CABL | 16172-16183C-16189-16223-16320-16519-73-150-152-195-198-263 | L3e2b | Sub-Saharan African | 190 | 1 | **16129**R-16172Y-16183A/C-16189Y-16223Y-**16262**Y-16320Y-16519Y-**73**G-150Y-152Y-195Y-198Y-**263**G-**(523-524)delAC**/non-del | ? | unknown | West Eurasian | A03 |
|  |  |  |  |  | 1 | **16129**R-16172Y-16183A/C-16189Y-16223Y-16320Y-16519Y-**52**Y-**73**G-150Y-152Y-195Y-198Y-**263**G-**(523-524)delAC**/non-del | ? | unknown | West Eurasian | C01 |
|  |  |  |  |  | 1 | **16129**R-16172Y-16183A/C-16189Y-16223Y-16320Y-16519Y-**73**G-150Y-152Y-195Y-198Y-**263**G-**(523-524)delAC**/non-del | ? | unknown | West Eurasian | D12 |
|  |  |  |  |  | 1 | 16172Y-16183A/C-16189Y-16223Y-16320Y-**16487**R-**16519**C-73R-150Y-152Y-195Y-198Y-**263**G-**610**Y | H | from member 24 or 27? | West Eurasian | B02 |
| B-1 | 16150-16182C-16183C-16189-16311-152-195-263-270 | H11 | West Eurasian | 95 | 1 | **16129**R-16150Y-16182A/C-16183A/C-16189Y-16311Y-**73**R-152Y-195Y-**263**G-270R-**(523-524)delAC**/non-del | ? | unknown | West Eurasian | F03 |
| B-3 | 16150-16182C-16183C-16189-16311-152-195-263-270 | H11 | West Eurasian | 95 | 0 | — | — | — | — | — |
| B-2 | 16150-16182C-16183C-16189-16311-152-195-263-270 | H11 | West Eurasian | 96 | 1 | **16129**R-16150Y-16182A/C-16183A/C-16189Y-16311Y-**73**R-152Y-195Y-**263**G-270R-**(523-524)delAC**/non-del | ? | unknown | West Eurasian | H06 |
| B-5 | 16150-16182C-16183C-16189-16311-152-195-263-270 | H11 | West Eurasian | 96 | 0 | — | — | — | — | — |
| B-10 | 16150-16182C-16183C-16189-16311-152-195-263-270 | H11 | West Eurasian | 95 | 0 | — | — | — | — | — |
| B-6 | 16150-16182C-16183C-16189-16311-152-195-263-270 | H11 | West Eurasian | 95 | 0 | — | — | — | — | — |
| B-9 | 16150-16182C-16183C-16189-16311-152-195-263-270 | H11 | West Eurasian | 95 | 0 | — | — | — | — | — |
| A-1 | 16168-16192-16256-16266-16294-16526-73-263-455delT-(523-524)insAC | U5a | West Eurasian | 91 | 0 | — | — | — | — | — |
| A-2 | 16168-16192-16256-16266-16294-16526-73-263-455delT-(523-524)insAC | U5a | West Eurasian | 94 | 2 | **16129**R-16168Y-16192Y-16256Y-16266Y-16294Y-16526R-**73**G-**263**G-455delT/non-del-**(523-524)delAC**/insAC | ? | unknown | West Eurasian | E09, E10 |
| A-3 | 16168-16192-16256-16266-16294-16526-73-263-455delT-(523-524)insAC | U5a | West Eurasian | 93 | 0 | — | — | — | — | — |
| A-4 | 16168-16192-16256-16266-16294-16526-73-263-455delT-(523-524)insAC | U5a | West Eurasian | 93 | 0 | — | — | — | — | — |
| A-6 | 16168-16192-16256-16266-16294-16526-73-263-455delT-(523-524)insAC | U5a | West Eurasian | 95 | 0 | — | — | — | — | — |
| A-5 | 16168-16192-16256-16266-16294-16526-73-263-455delT-(523-524)insAC | U5a | West Eurasian | 93 | 0 | — | — | — | — | — |
| Sample 11 | 16126-16294-16296-16304-16519-73-263 | T2b | West Eurasian | 287 | 0 | — | — | — | — | — |
| Sample 12 | 16126-16294-16296-16304-16519-73-263 | T2b | West Eurasian | 208 | 0 | — | — | — | — | — |
| CB-1 | 16129-16223-16391-16519-73-152-199-204-207-250-263-573insCs | I2 | West Eurasian | 375 | 0 | — | — | — | — | — |
| CB-2 | 16111-16223-16290-16319-16360-16362-97-(106-111)del-146-153-235-263 | A2 | Native American | 381 | 3 | 16129A-16223T-16391A-16519C-73G-152C-199C-204C-207A-250C-263G-573insCs | I2 | from sample CB-1 | West Eurasian | E07, E06, F05 |
|  |  |  |  |  | 1 | 16129A-16186Y-16223T-16391A-16519C-73G-152C-199C-204C-207A-250C-263G-573insCs | I2 | from sample CB-1 | West Eurasian | A08 |
| T3 | 16129-16224-16311-16519-16T-73-150-199-263-497-(523-524)insAC | K1a | West Eurasian | 343 | 1 | 16129A-73G-263G-(523-524)delAC | ? | unknown | West Eurasian | B03 |

Note: The following samples are siblings: donor 9 and BURDC; donor 10 and GREAC; HERJC and ANDGC; QUIDC and MAUWC; KIRGC and THOMC; CABO and CABL. The following samples are maternally related: samples A-1 to A-6; samples B-1 to B-3, B-5, B-6, B-9, B-10; samples 11 and 12. Donor 7 and sample 11 are current lab personnel and also contributed hair samples listed in Table 1 (samples 10 and 36, respectively). Sample T3 is a Jurkat T cell line. The contaminant in donor 9 was not determined in region 59-342 (due to the two heteroplasmic indels at site 56 and in region 515-524) and could not be sequenced with the primers used in this study; the contaminant in QUIDC was not determined in region 523-567 (due to the two heteroplasmic insertions in regions 515-524 and 568-573); further, the two contaminants in sample A-2 were not determined in region 455-513 (due to the two heteroplasmic indels at site 455 and in region 515-524).

^a^ The inferred sequence variation in the contaminant is in bold font. Haplogroup status and geographic origin of the contaminant DNA and the samples were estimated according to the available world mtDNA phylogeny [46-51,54]. A question mark was added to denote uncertainty of the classification. We scored the heterplasmic mutations (with the mutant allele present at >10% level) in the contaminated cells according to the sequencing electropherograms.
